# Enhanced reducing leachate pollution index through electrocoagulation using response surface methodology

**DOI:** 10.1016/j.heliyon.2024.e38134

**Published:** 2024-09-19

**Authors:** Fateme Ameli, Hassan Hashemi, Mohammad Reza Samaei, Esrafil Asgari, Mehran Mohammadian Fazli

**Affiliations:** a Department of Environmental Health Engineering, School of Public Health, Zanjan University of Medical Sciences, Zanjan, Iran; b Research Center for Health Sciences, Institute of Health, Department of Environmental Health Engineering, School of Health, Shiraz University of Medical Sciences, Shiraz, Iran

**Keywords:** Leachate pollution index, Electrocoagulation, Response surface methodology, Central composite design

## Abstract

Addressing the urgent need to effectively manage landfill leachate as a harmful flow for human health and the environment, this research investigates how electrocoagulation (EC) processes could alleviate the pollution potential of leachate. So far, no experimental study has been carried out on reducing the leachate pollution index (LPI) under the EC process. For this purpose, in this novel research, the LPI was utilized as a key metric to evaluate the efficiency of the treatment process. Central Composite Design (CCD) as a subset of Response Surface Methodology (RSM) was applied to enhance the LPI parameters decreasing percentage. The data were analyzed by analysis of variance and multivariate regression and 3D plots assessed variable interactions. Under optimal conditions, it showed removal of 97.48 % for COD, 91.42 % for BOD_5_, 98.52 % for N-NH_3_, and 91.6 % for TDS. Significant reductions were observed in 94.81 % TKN, 87.20 %, 82.80 %, 96.66 %, and 99.28 %, 99.18 %, and 96.56 % for TKN, Cl^−^, CN^−^, As, Cr, Zn, and Ni, respectively. Moreover, the kinetics of COD removal indicated that it follows a first-order model. Thus, based on experimental results, the LPI of raw leachate decreased from 38.06 to 7.22 (81 % decrease) under the EC treatment method. The study indicated that the EC treatment method successfully reduced leachate pollution and met the leachate discharge standard.

## Highlights

• The leachate pollution index was used as a criterion to assess the effectiveness of the EC process.
• Response surface methodology was utilized to model responses and optimize treatment processes.
• Three-dimensional response surface plots were interpreted to visualize the interactions between different parameters and their effects on the removal efficiency.
• Electrochemical mechanisms, reaction kinetics, and operating cost of the EC process were studied.
• The study reported significant removal of leachate pollution index parameters such as COD, BOD5, N-NH3, and TDS.

## Graphical abstract

**Figure:**
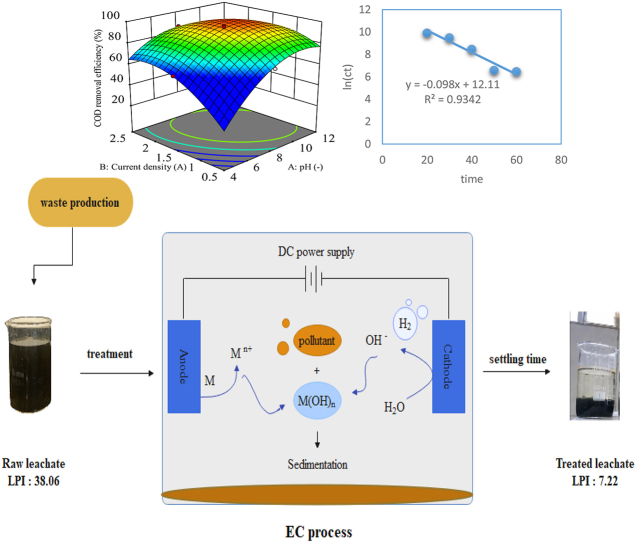

## 1 Introduction

Leachate is a toxic byproduct generated from landfills because of the interaction between the waste mass and the water entering the waste layers [1]. Engineered landfills represent a considerable advancement in waste management, with a focus on environmental protection. Exposure to landfill leachate can have adverse effects on human health due to the presence of cytotoxic and carcinogenic compounds such as heavy metals, PAHs, and ammonia. This can lead to oxidative stress which can cause kidney damage, cancer, and lower cognitive abilities, especially in children [2]. One of the main worries for leachate is the possibility of pollution caused by the movement of leachate from the underground soil to the surface and groundwater [3]. Unfortunately, the World Bank estimated in 2018 that at least one-third of Municipal Solid Waste (MSW) is not disposed of in an environmentally friendly manner and more than half of waste in Asia is openly dumped. At present, Asia has the largest number of dumpsites among the top fifty dumpsites in the world, with seventeen of them located on the continent [4]. Mishra et al. (2016) noted that improper management of leachate is among the main causes of soil, surface water, and groundwater contamination [5]. The research conducted by Hosseini Binabaj et al. delved into the presence of heavy metals in the soil and plants surrounding the landfill in Tehran. This investigation revealed that the soil and plants near the landfill had been tainted by heavy metals due to the impact of leachate [6]. Alavi Moghadam et al. (2009) research has demonstrated that the absence of appropriate infrastructure and facilities in developing countries has impeded the attainment of satisfactory waste collection, management, and disposal conditions. This mismanagement in the waste sector is reflected in Iran's pollution of surface and underground water, soil, and ecosystems due to landfill leachate [7]. The case study of Kooch et al. (2023), investigated the effect of landfill leachate on soil health indicators from different years in the Hyrkani forest area in northern Iran. Their findings showed that the absence of a leachate collection system and the use of unsanitary landfill sites have led to a decrease in the stability of soil grains, nutrient contents, microbial and enzyme activities, as well as the abundance of fauna and microflora in the region [8]. Pasalari et al. (2018) demonstrated that to ensure that landfill leachate is properly managed and treated before being released into the environment, it is important to investigate the characteristics and composition of landfill leachate and make informed decisions about which treatment methods to use based on its properties [9]. Therefore, to ensure the preservation of the environment and human well-being, it becomes paramount to thoroughly comprehend landfill leachate characteristics and determine the most appropriate approach for leachate management [10]. The Leachate Pollution Index (LPI) has emerged as a useful tool in assessing the degree of pollution potential caused by landfill leachate. This index was first introduced by Kumar and Alappat (2003) and has since been widely adopted in environmental studies [11]. The LPI consists of 18 parameters which are COD, BOD, N-NH_3_, pH, TDS, TKN, Zn, Pb, Cr, As, Hg, Ni, Cu, Fe, TCB, Phenolic Compounds, CN^−^, and Cl^−^ [12]. According to the Municipal Solid Wastes (Management and Handling) Rules of 2000, a recommended LPI value of 7.378 serves as a benchmark for permissible leachate levels that can be safely discharged into surface waters. The LPI allows for a standardized comparison of leachate quality between different landfill sites, which is crucial for the realization of the environmental effect of leachate on surrounding ecosystems. Also, it allows field professionals and policymakers to better understand leachate characteristics, enabling them to make informed decisions about leachate management [13]. Different methods were employed for leachate treatment including biological methods like activated sludge [14], physicochemical processes such as coagulation [15], membrane methods like reverse osmosis [16], and advanced oxidation processes [17]. The electrocoagulation (EC) has recently received attention for its cost-effectiveness, and adaptability to changes. It enhances wastewater quality without the need for chemical additives. There are various studies conducted that demonstrate the EC process could be effective in the removal of paper industry wastewater [18], dyes [19], antibiotics [20], landfill leachate [21], chromium [22], caustic wastewater [23], textile wastewater [24], natural water [25], medical wastewater [26]. This technique involves the utilization of electrical current to disrupt and combine pollutants. Its success in addressing intricate leachate compositions makes it a suitable option for leachate treatment. Moreover, these advantages support sustainable waste management practices, making it an appropriate option for tackling leachate pollution while minimizing environmental effects [27]. The EC process consists of three steps: 1. Electrode dissolution: When an electric current is applied, metal ions are released from the anode. Then the released Fe^2+^ is converted to Fe(OH)_3_ in the presence of O_2_. 3. Flocculation: Hydroxo-metal complexes aggregate with contaminants to form larger flocs that can be removed by sedimentation or flotation [28]. Meanwhile, the central composite design (CCD) using the response surface method (RSM) is a valorous tool for optimizing laboratory processes [29,30]. Therefore, the use of LPI for evaluation and EC for treatment process offers a complete way for controlling leachate pollution. As the LPI measures the pollution possibility, EC offers a feasible resolution for reducing that pollution. Based on the studies, it seems that the cautious investigation of landfill leachate characteristics using the LPI scale prior to implementing treatment methods has received little attention. Thus, the main objective of this study is to quantify the LPI parameters. Additionally, the study aims to mitigate the pollution risk of Barmshoor's landfill leachate from Shiraz, Iran by optimizing the EC process conditions. To assess the acceptable range of the leachate pollution index, the study takes into consideration the guidelines outlined in the Municipal Solid Wastes (Management and Handling) Rules of 2000. The study utilizes the RSM to design, conduct, analyze, optimize, and interpret experimental data. In addition, the kinetics and rate constant for COD removal were analyzed.

## 2 Material and methods

### 2.1 Leachate collection and characterization

Leachate sampling was done in the summer of 2023 from a drain of the sanitary landfill situated in Shiraz, Iran. Samples of leachate were gathered in a 20-liter container. The container was transferred to the Environmental Health Engineering Laboratory of Shiraz Health Faculty and was kept at 4 °C until analysis. Measurements of LPI parameters (COD, pH, BOD_5_, N-NH_3_, TDS, TKN, Cl^−^, CN^−^, and heavy metals of (As, Hg, Cr, Pb, Zn, Ni, Cu, Fe)) carried out according to what was proposed by APHA procidures. COD was determined using the closed reflux colorimetric method by spectrophotometer (HACH DR5000, HACH Company, Loveland, CO, USA). BOD_5_ was determined using Method 5210B. For this purpose, the dissolved oxygen (DO) was measured using a DO meter (model HQ11D HACH Company, Loveland, CO, USA). pH was measured by a portable pH meter (SANXIN comb. pH, SanXin Instrumentation Inc, Shanghai, China). TKN was measured utilizing boric acid, sodium hydroxide, indicators, and titration with HCl solution after distillation of steam with a Unit Pro-Nitro-S JP SELECTA device. N-NH_3_ was analyzed by the phenate method. TDS was measured using the gravimetric method. CL^−^ and CN^−^ were determined using the Ion chromatography (IC). Also, heavy metals were determined by ICP-MASS.

### 2.2 Chemicals

Chemicals utilized in this research, including sulfuric acid (H₂SO₄), sodium hydroxide (NaOH), potassium dichromate (K₂Cr₂O₇), silver sulfate (Ag₂SO₄), and the Nessler reagent, were procured from Merck (Darmstadt, Germany) and Sigma (Ronkonkoma, NY, USA).

### 2.3 Experimental procedure

**Fig. 1:**
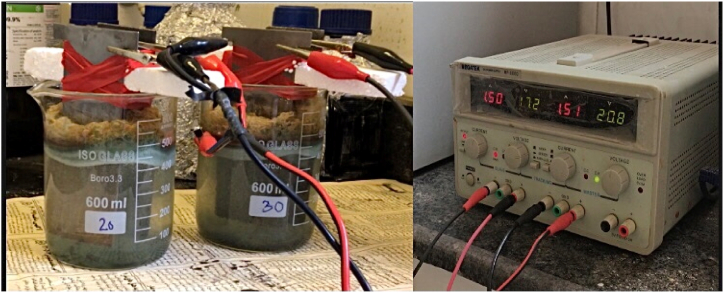
The schematic of the EC process includes the power supply, and iron electrodes immersed in the sample.

The EC process was carried out in batch mode with a sample volume of 600 mL in a glass reactor. As shown in Fig. 1, iron electrodes with dimensions of 10 cm × 5.0 cm and 1.0 mm in thickness were used as cathode and anode in the EC process, providing an effective surface area of 7.0 cm × 5.0 cm × 1.0 mm. To adjust the current density in the electrochemical cell, the current supply voltage of 3A/5V (MEGATEK, MP3003 D, Villeneuve le Roi, France) was utilized. The variables of pH, current density, inter-electrode distance, and electrolysis time, which were determined by reviewing the literature and pre-experiments. The initial pH sample was adjusted using HCL and NaOH solutions. Following each process, sedimentation was allowed to occur for 2 h. The treatment process' supernatant was then utilized to analyze physical and chemical characteristics. In order to improve the quality of the data, the supernatant from each process was centrifuged before analyzing the samples to prevent interference caused by the clots formed during the coagulation process.

### 2.4 Model development

The CCD, RSM was carried out using Design Expert-12 software to ascertain the ideal state and mathematical modeling of the EC process [24]. Therefore, COD, BOD_5_, N-NH_3_, and TDS removal efficiency were used as responses in lowering the leachate pollution potential. CCD involves using statistical techniques to develop a regression model based on quantitative data. This model relates independent variables to response variables and results in a decrease in the required number of experimental runs. The rest of the parameters including pH, TKN, CL^−^, Cr, Pb, Hg, As, CN^−^, Zn, Ni, Fe, and Cu were measured under optimal conditions for calculating the LPI of treated leachate. The values and levels of the factors, for each process were considered, as shown in Table 1. Therefore, 30 experiments with 6 central points were designed for the EC process.

**Table 1:** Factors and levels of the independent variables in the EC process.

Input Variable	Symbol	Coded Values				
		−2	−1	0	+1	+2
		Uncoded Values				
pH	A	4	6	8	10	12
Current Density (A/m^2^)	B	0.5	1	1.5	2	2.5
Inter-Electrode Distance (cm)	C	1	1.5	2	2.5	3
Electrolysis Time (min)	D	20	30	40	50	60

### 2.5 Leachate pollution index (LPI)

This study evaluated how well the EC treatment method reduce the pollution levels in leachate. As a result, the values of the parameters in the raw and treated leachate were measured. Then the three sub-indices of the LPI and the overall LPI were calculated. The formula below can be used to calculate each sub-index (Eq. (1)):

(1) $$\text{LPI}=\frac{\sum_{i=1}^{m}w_{i}\;p_{i}}{\sum_{i=1}^{m}w_{i}}$$

Therefore, the total LPI can be calculated (Eq. (2)):

(2) LPI = 0.232 LPI_or_ + 0.257 LPI_in_ + 0.511 LPI_hm_

To do this, the weight assigned to each variable (W_i_), the sub-index score for each variable (P_i_), and the number of leachate pollutant variables used in constructing the LPI (n) need to be considered. The criteria outlined by Kumar and Alappat (2005) were followed in adopting the W_i_ and P_i_ [31,32].

### 2.6 Operating cost of the EC process

The costs involved in operating the EC process are included in various categories. Apart from materials such as electrodes and electricity expenses, there are also costs related with labor, retention, sludge removal, and disposal, as well as fixed costs. These fixed costs do not depend on the type of electrode material being employed.

(3) Energy = VIt/VR

According to Eq. (3), (V) is related to the average voltage of the EC process, (I) represents the value of the electrical current density, (t) stands for reaction time, and VR is the wastewater volume, respectively.

(4) Operating Cost = aC_energy_ + bC_electrode_ + D

As demonstrated in Eq. (4), it can see that (a) represents the cost of the electrical energy per kilowatt-hour in dollars, (b) is the electrode material's cost per kilogram in dollars, (D) stands for the cost of utilized chemicals, and (C_energy_) and (C_electrode_) correspond to the utilization of energy and electrodes, respectively. Eq. (3) is used to calculate C_energy_.

(5) C_electrode_ (kg/m^3^) = ItM/nFV

In Eq. (5), M is Fe's molecular mass (55.85gmol^−1^), n demonstrates the number of electrons transported Fe (Z = 2), F is the constant of Faraday (96,487 C mol^−1^), and V is the wastewater volume [33].

## 3 Results and discussion

### 3.1 Physical and chemical properties of leachate

Understanding the composition of leachate is crucial for evaluating its environmental impact, determining the appropriate treatment method, and preventing the pollution of resources [34]. The data obtained from the analysis of the leachate characteristics can be found in Table 2. The leachate demonstrated a COD value of 28851 mg/L. Leachate sampling was done in the summer season, which is why the COD value was high. A similar result was also obtained by Hoai et al. (2021). They observed a significant increase in the concentration of BOD_5_ and COD, 5 to 8 times higher in the leachate during the dry season compared to the rainy season [35]. Also, the ratio of BOD_5_/COD of raw leachate was determined at 0.3. According to Metcalf and Eddy. (1991), wastewater can be efficiently biodegradable when the BOD_5_/COD ratio falls within the range of 0.4–0.8. Therefore, a BOD_5_/COD ratio of 0.3 indicates that landfill leachate has a moderate potential for biodegradation [36]. The mild alkalinity (8) of leachate suggests that the leachate has finished its acidification period and is now in the methanogenic phase [37]. Moreover, when the leachate ages and moves towards the alkaline phase, organic nitrogen breaks down into ammonium N, and the level of ammonia nitrogen rises. Similarly, the analysis results revealed a high amount of ammonia at 1610 mg/L [38]. It seems that the N-NH_3_ measurements obtained from Barmshoor leachate are still higher compared to the findings of Rookesh et al. (2022). According to their study they reported a value of 486 mg/L, for N-NH_3_ in leachate collected from a landfill site, in Yasuj, Iran [39]. Furthermore, Sharafinasab et al. (2021) highlighted the significance of 44.07 obtained from a leachate sample taken in Saravan town, Iran [40]. However, the obtained result of the N-NH_3_ value in some studies is higher. Zaki et al. (2022) found a high amount of ammonia in the value of 3317 mg/L in their results [41]. Also, according to research conducted by Ishak et al. (2018), the amount of ammonia in the raw leachate was 2700 mg/L [42]. It was observed that the leachate had a low quantity of heavy metals. However, when comparing the metals, Zinc exhibited a higher value of 6.16 while Iron recorded the highest value of 14.7 mg/L. In a research conducted by Beinabaj et al. (2023), the levels of heavy metals in the leachate of the Aradkoh landfill situated in the Kahrizak area of Tehran, Iran were measured. The findings indicated that iron had the highest concentration among other metals that were studied. Specifically, two separate samples revealed iron concentrations of 22.94 and 17.01 mg/L, respectively [6]. Therefore, the landfill leachate produced from the Barmshoor landfill is at a moderate age and alkaline phase. Hence, according to the leachate characteristic, biological treatment processes can not be effective for this complex compound. However, considering the high TDS and ammonia in the leachate, coagulation methods, especially EC, seem to perform well in reducing the leachate pollution potential.

**Table 2:** Raw leachate characterization in this study.

Parameter	Value	unit
pH	8 ± 0.08	–
COD	28851 ± 280.49	mg/L
BOD_5_	9560 ± 82.25	mg/L
TDS	34580.9 ± 74.19	mg/L
Ammonical nitrogen	1610 ± 7.54	mg/L
TKN	2120 ± 3.29	mg/L
Chlorides	12500 ± 12.25	mg/L
Cyanide	0.05 ± 1.4	mg/L
Chromium	0.42 ± 0.3	mg/L
Arsenic	0.15 ± 0.2	mg/L
Mercury	0.001 ± 0.3	mg/L
Lead	0.001 ± 0.31	mg/L
Zinc	6.16 ± 0.5	mg/L
Nickel	0.553 ± 0.4	mg/L
Copper	0.001 ± 0.1	mg/L
Iron	14.7 ± 0.6	mg/L
Manganese	0.001 ± 0.3	mg/L
Cadmium	0.001 ± 0.24	mg/L
Leachate pollution index (LPI)	38.06	–

### 3.2 Experimental results and data analysis

The study used ANOVA and response surface plots to analyze the effects of different variables. Also, statistical indices such as R^2^, adjusted R^2^, predicted R^2^, and lack-of-fit were utilized for the quality assessment of the response models and their fit [43]. Table 3 shows the ANOVA results of responses in the EC process. The importance and significance of terms and the model can be assessed by analyzing the P-value and F-value. P-values less than 0.05 indicate the term's effect on the model and its significance. When the P-value is smaller, it means that the coefficients associated with it are more significant in predicting the outcome. Also, a higher F-value enhances the model's validity. Repeating central points in experiments helps calculate errors and identify a lack of fit [[44], [45], [46]]. As shown in Table 3, the linear effect of pH with a high F-value has the highest impact on the removal of COD, BOD_5_, and TDS. The model's high F-value for COD (35.63) and BOD_5_ (54.64) suggests that the models have enough validity for both analyzing results [47]. With 95 % confidence, the linear terms of pH, C.D, and Distance significantly influence N–NH3 removal, with P-values of <0.0001, <0.0001, and 0.0003, respectively. According to linear terms, C.D has more statistical significance than time in COD removal. In the study conducted by Faraj et al., in 2024, they found similar results that the C.D variable showed more influence on the COD removal and energy consumption than the time variable [48].

**Table 3:** ANOVA of the four responses in the EC process to treatment of leachate.

Source	COD			BOD_5_			N-NH_3_			TDS		
	Sum of Squares	F-value	P-value	Sum of Squares	F-value	P-value	Sum of Squares	F-value	P-value	Sum of Squares	F-value	P-value
Model	3135.54	35.63	<0.0001	1534.56	54.64	<0.0001	2236.87	17.71	<0.0001	5062.25	127.83	<0.0001
A-pH	1013.35	161.23	<0.0001	593.72	295.94	<0.0001	391.40	43.39	<0.0001	4485.95	453.12	<0.0001
B-C.D	288.91	45.97	<0.0001	49.85	24.85	0.0002	908.72	100.75	<0.0001	240.29	24.27	<0.0001
C-Distance	535.15	85.14	<0.0001	108.84	54.25	<0.0001	190.52	21.12	0.0003	335.70	33.91	<0.0001
D-Time	153.27	24.39	0.0002	129.87	64.74	<0.0001	23.80	2.64	0.1251	0.3128	0.0316	0.8603
AB	121.61	19.35	0.0005	177.36	88.40	<0.0001	4.71	0.5221	0.4811			
AC	85.89	13.66	0.0022	55.47	27.65	<0.0001	155.25	17.21	0.0009			
AD	0.3630	0.0578	0.8133	14.96	7.46	0.0155	10.24	1.14	0.3035			
BC	7.33	1.17	0.2972	16.46	8.21	0.0118	88.55	9.82	0.0068			
BD	2.97	0.4721	0.5025	37.91	18.90	0.0006	19.89	2.21	0.1582			
CD	7.85	1.25	0.2812	0.2475	0.1234	0.7303	53.44	5.92	0.0279			
A^2^	593.05	94.36	<0.0001	211.11	105.23	<0.0001	201.93	22.39	0.0003			
B^2^	397.35	63.22	<0.0001	34.75	17.32	0.0008	238.43	26.43	0.0001			
C^2^	18.38	2.92	0.1078	7.91	3.94	0.0657	5.67	0.6284	0.4403			
D^2^	147.46	23.46	0.0002	130.41	65.00	<0.0001	3.5	0.3877	0.5428			
Lack of Fit	72.62	1.68	0.2959 0.2959 Not sign.	20.11	1.01	0.5313 0.5313 Not sign.	107.04	1.89	0.2492 0.2492 Not sign.	169.74	0.5457	0.8479 0.8479 Not sign.
C.V %	2.97			1.9			3.53			4.14		
R^2^	0.9708			0.9808			0.9430			0.9534		
Adj R^2^	0.9436			0.9628			0.8897			0.9459		
Pred R^2^	0.8608			0.9168			0.7229			0.9360		

The COD model effectively demonstrates the relationship between the response and variables with an F-value of 35.63 and a P-value less than 0.0001 [48]. All linear terms, except for the CD and C^2^ with a P-value of 0.7303, and 0.0657 significantly influence the BOD_5_ removal. The N-NH_3_ removal response with an F-value of 17.71 and P-value <0.0001 demonstrates significance. For TDS removal efficiency, the pH, C.D, and distance variables significantly affect by P-values in <0.0001. Another way to confirm the accuracy of the suggested models is by checking the P-value of the lack of fit. Based on the results, the P-values of lack of fit are 0.2959, 0.5313, 0.2492, and 0.8479 for COD, BOD_5_, N-NH_3_, and TDS, respectively. The non-significant value of lack of fit demonstrates the model's fit with the experimental data. Its also confirms that the central points were repeated correctly in the experiments [49]. The coefficient of variation (C.V) used to determine the relative dispersion of the experimental data from the prediction of models. It is calculated as the ratio of the standard error of the estimates to the mean of the responses. The C.V of the model should be below 10 % that considered as reproducible model [50]. According to Table 3, the C.V values are 2.97 for COD, 1.9 for BOD_5_, 3.53 for N-NH_3_, and 4.14 for TDS that demonstring the high repeatability of the model of the responses. The R^2^ values of the correlation coefficient in the present study for COD, BOD_5_, N-NH_3_, and TDS were higher than 0.90 in the range of (0.9430–0.9808). Therefore, models demonstrate a strong correlation between factors and responses, indicating excellent validity [51]. According to the Sum of Squares analysis, it is evident that linear terms have the most significant effect on COD removal efficiency of 63.46 %. quadratic terms rank second with an influence of 36.86 % on the response. Interaction terms have the least effect 7.18 %. In BOD_5_ removal efficiency, the order of influence is as follows: linear terms have the highest impact at 57.49 % (38.68 % for A, 3.24 % for B, 7.09 % for C, and 8.46 % for D), followed by quadratic terms at 25.03 % (13.75 % for A^2^, 2.26 % for B^2^, 0.51 % for C^2^, and 8.49 % for D^2^), and interaction effects at 19.70 % (11.55 % for AB, 3.61 % for AC, 0.97 % for AD, 2.47 % for BC, 0.11 % for BD). For N-NH_3_ the influence of linear terms was 67.70 %, quadratic terms were 14.84 %, and interaction terms were 24.88 %.

### 3.3 Equations of regression model

The regression equations derived from the experimental models for each response in the EC processes are presented in Eqs. (6)–(9). The software recommended quadratic models for all the responses, except for TDS removal, for which linear models were selected. The positive and negative signs indicate direct and inverse effects in front of the coefficients in the equations. According to Eqs (6)–(9), the pH, C.D, and time variables had a positive influence however, the inter-electrode distance had a negative effect on the removal of COD, BOD_5_, and TDS. Also, pH and the C.D had a positive effect and the electrolysis time and the inter-electrode distance had a negative effect on the removal of N-NH_3_ response. It can be inferred that the quadratic conditions of all the variables have negative effects on the responses, except for the response of BOD_5_ removal. In this case, the variables of pH, current density, and electrolysis time have a negative effect whereas the inter-electrode distance has a positive effect. The interaction between the variables in the responses varies. In regards to COD removal efficiency, the negative interaction effects are observed in pH-C.D, pH-inter-electrode distance, and pH-time. On the other hand, positive interaction effects are demonstrated in C.D-inter-electrode distance, C.D-time, and inter-electrode distance-time.

COD removal (%) = 93.68 + 6.50 A + 3.47 B - 4.72 C + 2.53 D - 2.76 AB - 2.32 AC - 0.1506 AD + 0.6769 BC + 0.4306 BD + 0.7006 CD - 4.65 A^2^ - 3.81 B^2^ - 0.8186C^2^ - 2.32 D^2^ (Eq. 6)

(Eq.7) BOD_5_ removal (%) = 78.87 + 4.97 A + 1.44 B - 2.13 C + 2.33 D - 3.33 AB + 1.86 AC - 0.9669 AD + 1.01 BC - 1.54 BD + 0.1244 CD - 2.77 A^2^ - 1.13 B^2^ + 0.5370C^2^ - 2.18 D^2^

(Eq.8) N-NH_3_ removal (%) = 90.21 + 4.04 A + 6.15 B - 2.82 C - 0.9958 D - 0.5425 AB - 3.11 AC -0.8000 AD + 2.35 BC + 1.11 BD - 1.83 CD - 2.71 A^2^ - 2.95 B^2^ - 0.4546C^2^ - 0.3571 D^2^

(Eq.9) TDS removal (%) = 76.07 + 13.67 A + 3.16 B - 3.74 C + 0.1142 D

### 3.4 Main effect of factors

**Fig. 2:**
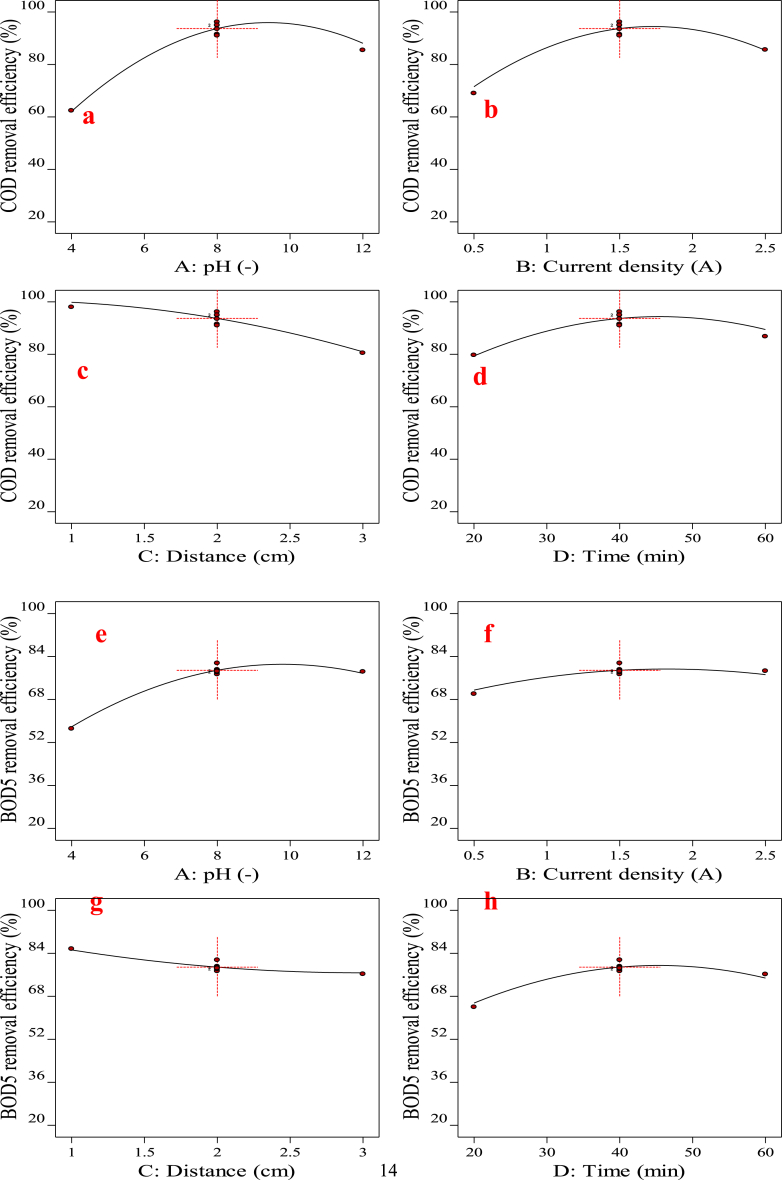

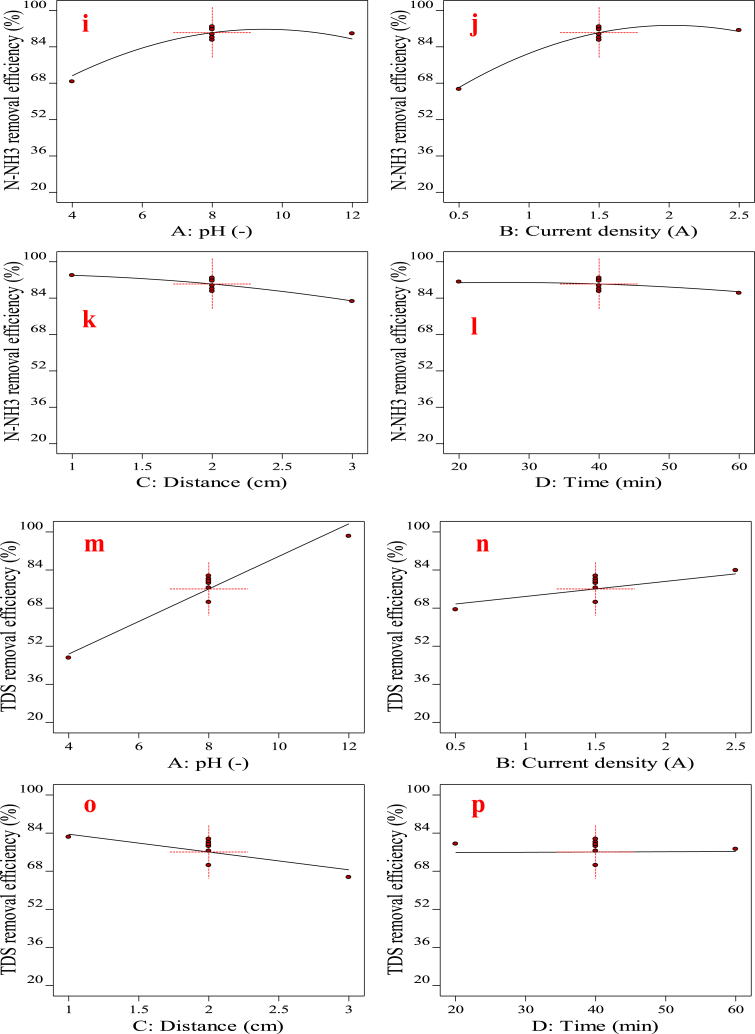
Main effect of factors on responses: (a) pH, (b) Current density, (c) Distance and (d) Time for COD removal, (e) pH, (f) Current density, (g) Distance and (h) Time for BOD_5_ removal, (i) pH, (j) Current density, (k) Distance and (l) Time for N-NH_3_ removal, (m) pH, (n) Current density, (o) Distance and (p) Time for TDS removal, respectively.

According to Fig. 2(a)–(d), for COD it is suggested that by maintaining a pH level of 8.0 and a current density of 1.5 A/m^2^, the optimal condition for the lowest inter-electrode distance would be achieved. It may be due to the formation of more gas bubbles in a narrower inter-electrode distance. This leads to an increased possibility of collisions between coagulants and pollutants, resulting in enhanced treatment efficiency due to turbulence formation [52]. In the case of BOD_5_, the current density does not significantly affect the removal efficiency. Increasing the electrolysis time to 40 min improves the removal efficiency, but beyond 40 min, the BOD_5_ removal decreases, as demonstrated in Fig. 2(f) and (h). Based on Fig. 2(j) and (l), the electrolysis time does not have a significant effect, and increasing the current density up to 1.5 A/m^2^ results in higher N-NH_3_ removal efficiency. Moreover, greater distance leads to decreased removal, as indicated in Fig. 2(k). TDS in Fig. 2(m) and (p), display the curves are completely linear due to the linear model and have a steeper slope than other responses. It is observed in Fig. 2(m) and (n), that increasing the pH and current density leads to increased removal. However, as expected, increasing the inter-electrode distance decreases TDS removal, as shown in Fig. 2(o).

### 3.5 The effect of the variables using 3D surface plots

**Fig. 3:**
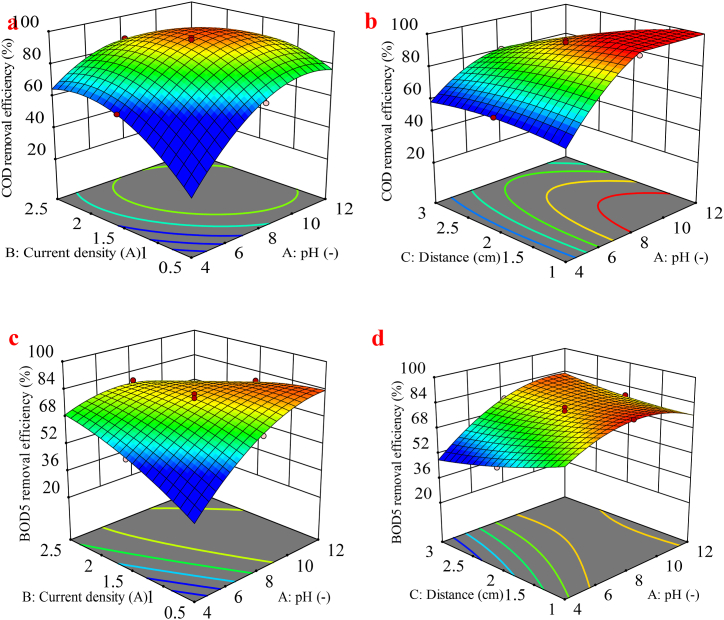

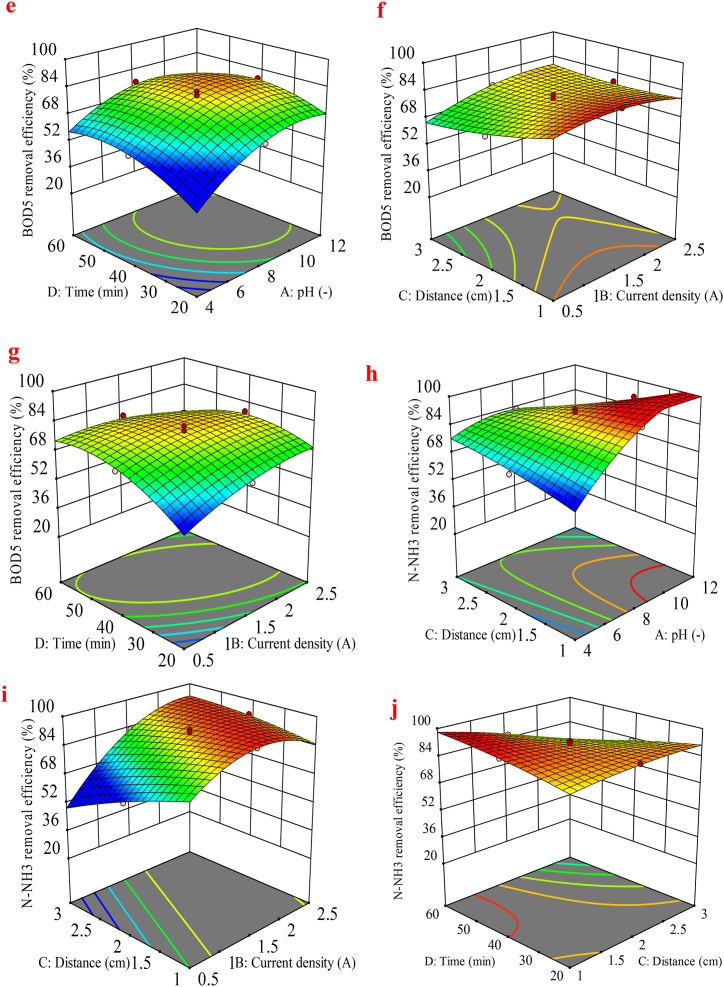
3D surface plots presenting the effect of factors (a) pH and Current density, (b) pH and Distance on the COD removal, (c) pH and Current density, (d) pH and Distance (e) pH and Time (f) Current density and Distance (g) Current density and Time, (h) pH and Distance on the BOD_5_ removal, (i) Current density and Distance (j) Distance and Time on the N-NH_3_ removal for EC process.

Three-dimensional response surface plots demonstrate the visualization of the interactions between these parameters on the study responses (COD, BOD_5_, N-NH_3_, and TDS) for the EC process in Fig. 3(a)–(j). Results show that the interaction of variables does not have a significant effect on COD removal, except for the AB and AC interactions. Fig. 3(a) and (b), illustrate the interaction effect of pH-current density and pH-distance on COD removal efficiency. The data indicate that within the current density range of 1.5–2.0 A/m^2^ and a pH range of 8.0–10, there is a marked increase in COD removal efficiency compared to other conditions. Additionally, analysis of the interaction between pH and electrode distance demonstrates that optimal efficiency is achieved when the pH is alkaline and the electrode distance is less than 2.0 cm. Thus, COD removal efficiency is directly correlated with pH and inversely correlated with electrode distance. One reason for the enhanced removal of COD with increasing pH is the augmented formation of metal hydroxides on iron electrodes, which improves the coagulation process and subsequently enhances COD removal efficiency [53]. Also, this case could be explained by the production of hydrogen gas at the cathode and the flourish of hydroxyl ions in the alkaline solution, leading to improved removal efficiency [53,54]. Current density is a fundamental parameter in EC, significantly influencing the formation and size of flocs. As shown in Fig. 3(a), an increase in current density generally enhances the removal efficiency of COD. The electrochemical reaction involves three essential processes: electrolytic reactions taking place on the electrodes' surface, the generation of coagulants in the liquid phase, and the adsorption of soluble and colloidal pollutants onto the coagulants, followed by their removal through settlement or flotation. The reactions that take place during the electrochemical process when using an iron electrode in the anode and cathode generally involve two mechanisms that result in the production of coagulants, shown in Eqs. (10), (11), (12), (13), (14), (15), (16), (17)) [55,56].

First mechanisms:

(10) Anode: Fe_(s)_ → Fe^2+^_(aq)_+ 2e^−^

(11) Fe^2+^_(aq)_ + 2OH^−^_(aq)_ → Fe(OH)_2(s)_

(12) Cathode: 2H_2_O_(l)_ + 2e^−^ → 2OH^−^_(aq)_ + H_2(g)_

(13) Overall reaction: Fe_(s)_ + 2H_2_O_(l)_ → Fe(OH)_2(s)_ + H_2(g)_

Second mechanisms:

(14) Anode: 4Fe_(s)_ → 4Fe^2+^_(aq)_+ 8e^−^

(15) 4Fe^2+^_(aq)_ + 10H_2_O_(l)_ + O_2(g)_ → 4Fe(OH)_3(s)_ + 8H^+^_(aq)_

(16) Cathode: 8H^+^_(aq)_ + 8e^−^→ 4H_2(g)_

(17) Overall reaction: 4Fe_(s)_ + 10H_2_O_(l)_ + O_2(g)_ → 4Fe(OH)_3(s)_ + 4H_2(g)_

According to the mechanisms of the EC process, anionic reactions produce Fe^2+^, Fe^3+^, H^+^, and Fe(OH)_2_, while cathodic reactions yield hydrogen gas and hydroxyl ions. The iron ions produced in the anode react with the hydroxyl ions produced during the water revival in the cathode to form a gelatinous suspension of hydroxyl iron Fe(OH)_n_. Pollutants, including colloids and solubles, get trapped and are removed when heavy clots of iron hydroxide are deposited under electrostatic attraction mechanisms. Light particles also rise to the top of the wastewater due to their interaction with the hydrogen gas bubbles formed at the cathode, and they get eliminated. Therefore, with the increase in current density, the amounts of iron ions and hydroxyl ions produced will increase, leading to a higher possibility of clot formation [57,58].

However, Fig. 3(a) shows that when the current density exceeds 2.0 A/m^2^, the COD removal efficiency decreases. This reduction is likely due to the higher oxygen generation from the anode may lead to a reduction in Fe(OH)_3_ formation due to the contestation between anode dissolution and bubble formation [59]. According to Fig. 3(c), optimal BOD removal efficiency can be achieved at an alkaline pH range of 10–12, even with low current densities. This indicates that increasing the pH reduces the need for high electrical energy consumption. Fig. 3(f), illustrates that the distance between the electrodes has an inverse relationship with removal BOD as the distance decreases, removal efficiency increases. The desired efficiency is achieved when the distance between electrodes is minimized, especially in alkaline pH conditions (10–12), as demonstrated in Fig. 3(d). In Fig. 3(e), the interrelationships suggest optimal efficiency at the alkaline phase of pH and within an electrolysis time range of 30–50 min. Additionally, Fig. 3(f) emphasizes that at various current densities, better removal efficiency is obtained with smaller electrode distances. Finally, Fig. 3(e) and (g), demonstrate that both increased current density and extended time improve removal efficiency, indicating a direct relationship between these factors and removal efficiency. Moreover, the decrease in efficiency in COD, and BOD_5_ in Fig. 3(b) and (f) when the distance between electrodes increases may be because of the stronger electric field in the EC process due to the minus distance. This accelerates the speed of the charged particles toward the electrodes, resulting in stronger flocculation and settling of colloidal particles [54]. Fig. 3(h)–(j), demonstrate that N-NH_3_ removal efficiency improves with increasing pH and decreasing electrode distance. Effective removal efficiency is observed when the electrode distance is approximately 2.0 cm or less. However, when the electrode distance exceeds 2.0 cm, the removal efficiency declines, highlighting the significant inverse relationship between electrode distance and removal efficiency. Additionally, the distance between the electrodes influences current density and removal efficiency over time. The interactions in Table 3, show AC, BC, and CD terms notably influence the impact on the N-NH_3_. In Fig. 3(i), it can be considered that by raising the rate of current density, the efficiency is upgraded. Meanwhile, the N-NH_3_ removal efficiency within applying a current density of 0.5, and 1.0 A/m^2^ are 79.96 % and 89.54 %, respectively with a consistent pH of 8 and distance of 1 cm, as shown in Fig. 3(i). In the study conducted by Hamid et al., in 2020, an increase in current density and zeolite dose resulted in a higher removal efficiency of ammonia nitrogen from Malaysian landfill leachate, corroborating the findings of this research [60].

### 3.6 Optimization of the process and verification

One of the practical options of RSM is to optimize variables to achieve the best and most efficient test conditions. According to the optimal conditions, pH 8, 1 A/m^2^ of current density, 0.5 cm of enter-electrode distance, and 48 min of electrolysis time, the following removal efficiencies were obtained: 97.48 % for COD, 91.42 % for BOD, 98.52 % for N-NH_3_, and 91.6 % for TDS. Also, in this condition the removal efficiencies for other parameters of the LPI were as follows: 94.81 % for TKN, 87.20 % for Cl^−^, 82.80 % for CN^−^, 99.28 % for Cr, 96.66 % for As, 99.18 % for Zn, and 96.56 % for Ni. The value of the other parameters of leachate such as Pb, Hg, and Cu, were below the detection limit. Due to employing iron electrodes in the EC process, the iron concentration in leachate rose from 14.7 to 586.34 mg/L in the leachate treatment effluent. In Table 4, the comparison is made between the LPI in the raw sample and treated leachate. The results show that the EC process effectively reduced the leachate pollution potential. The LPI decreased from 38.06 to 7.22 after the EC process, meeting the Municipal Solid Wastes (Management and Handling) Rules of 2000 requirement for discharging treated leachate into the environment. These findings underscore the efficacy of EC processes, particularly when optimized using RSM, in achieving substantial removal efficiencies across a spectrum of wastewater pollutants, thereby contributing to environmental sustainability and compliance with regulatory standards.

**Table 4:** The LPI for raw and treated leachate by EC process.

Leachate type			Raw			treated		
Index	Parameter	W_i_	Value	P_i_	W_i_P_i_	Value	P_i_	W_i_P_i_
. LPI_or_	COD	0.267	28851	85	22.69	726	11	2.93
	BOD_5_	0.263	9560	65	17.09	350	10	2.63
	Phenolic compounds	0.246	–	–	–	–	–	–
	Total coliform bacteria	0.224	–	–	–	–	–	–
LPI_in_	pH	0.214	8	3	0.642	6.87	6	1.28
	TKN	0.206	2120	75	15.45	110	6	1.23
	N-NH_3_	0.198	1610	100	19.8	23.8	6	1.18
	TDS	0.195	34580.9	80	15.6	2903	8	1.55
	Cl^−^	0.187	12500	100	18.7	1600	12	2.24
LPI_hm_	Cr	0.125	0.42	5	0.625	0.003	5	0.625
	Pb	0.123	0.001	5	0.615	0.001	5	0.615
	Hg	0.121	0.001	5	0.605	0.001	5	0.605
	As	0.119	0.15	5	0.595	0.005	5	0.595
	CN^−^	0.114	0.05	5	0.57	0.0086	5	0.57
	Zn	0.110	6.16	6	0.66	0.05	5	0.55
	Ni	0.102	0.553	5	0.51	0.019	5	0.51
	Cu	0.098	0.001	5	0.49	0.001	5	0.49
	Fe	0.088	14.7	5	0.44	586.34	5	0.44
Overall LPI			38.06			7.22		

### 3.7 Kinetics of COD removal

kinetic analyses conducted for the EC process as suggested in the existing literature [61]. The reaction kinetics of the COD removal can be displayed by the pseudo-first-order and pseudo-second-order in Eqs.(18) (18), (19)).

(18) Ln C_0_/C = kt_0_

Where the rate constant is shown by k (min^−1^), the initial COD concentration is denoted as C_0_ (mg/L), and the COD concentration at time 't' is represented as C (mg/L).

(19) t/q_t_ = 1/k_2_ qe^2^ + t/qe

Where the q_t_ displays the quantity of pollutant that is removed per unit mass of adsorbent at a given time 't' (mg/g), q_e_ represents the pollutant removal efficiency per unit mass of adsorbent at equilibrium (mg/g), k_2_ is the rate constant of pseudo-second-order adsorption (g/mg·min), and t is the time (min).

Table 5, displays the comparison between the results of the pseudo-first-order and pseudo-second-order kinetic models for COD removal under optimal conditions in landfill leachate treatment. It can be seen that the COD removal follows a first-order kinetics model, with a R^2^ value of 0.934, and a reaction rate constant of 0.049 min. In the study conducted by Patel et al. (2021), it was demonstrated that chromium removal follows a first-order reaction during kinetic analysis in the current density range of 13.77–68.87 A/m^2^ using copper electrodes in the electrocoagulation system [61]. Also, Nandi et al. (2013) found that the removal of pararosaniline hydrochloride dye follows a first-order reaction [62]. These studies confirm current study results.

**Table 5:** The results of the kinetic models of COD removal.

Parameters	First - order	Second - order
R^2^	0.934	0.8587
K	0.049	2 × 10^−6^

### 3.8 Calculation of the operating costs for the EC process

Despite the unique characteristics of the EC method, such as not requiring chemical coagulants, one limitation of this method is its operating costs, especially on a larger scale. Moreover, as the demand for sustainable wastewater treatment solutions increases, understanding the cost implications of EC will be crucial for its adoption in various industries. Therefore, estimating the operating cost of the EC method is urgent to assess its economic efficiency [63]. It is found that the total energy that electrodes consumed was 0.00736 $/kw.hour^−1^ for Fe – Fe electrodes. The total expended cost for the chemical usage was 2.64 $. Thus, the sum of the operating cost was 2.66 $/m^3^.

### 3.9 Comparison of results from Previous literature studies with the present study

Based on the data in Table 6, electrochemical processes have proven highly effective in removing COD and other pollutants from various types of wastewater. For instance, the highest COD removal efficiency reported in the current study was 97.48 %. Similarly, COD removal efficiencies of 92 % were reported for dairy wastewater and industrial area wastewater, respectively. These results indicate that electrochemical processes, particularly when combined with other methods such as adsorption and photocatalysis, can be highly efficient in wastewater treatment.

**Table 6:** Studies conducted in the field of EC process.

Effluent Description	processes	Response Variable	Removal efficiency (%)	Reference
Aged landfill leachate	EC based continuous-flow reactor	COD, TOC, BOD_5_, N-NH_3_, colour, and turbidity	59, 64, 55, 27, 59, and 86	[53]
landfill leachate	EC	COD, BOD_5_, and turbidity	90, 92.3, and 99.6	[64]
Stabilized landfill leachate	activated persulfate process and EC	COD	88.67	[65]
landfill leachate	EC with solar photo Fenton	COD and colour	90 and 91	[66]
Petroleum industry effluent	EC associated with adsorption	COD, calcium and Strontium	52, 88 and 72	[67]
Industrial estate wastewater	EC with added electrolyte and H_2_O_2_	COD	92	[68]
Dairy wastewater	EC-assisted adsorption	COD and Turbidity	92 and 97	[48]
hazardous waste landfill leachate	EC	COD, cadmium, zinc, phenolic compounds, lead, TOC, and colour	90, 58.1, 63.6, 42.4, 52.5, 54.7, and 84	[21]
landfill leachate	EC	COD, BOD, N-NH_3_, and TDS	97.48, 91.42, 98.52, and 91.6	Present Study

### 3.10 Challenges and recommendations for future studies

The EC process method has several limitations, similar to other treatment processes. One of the things that should be mentioned is the formation of foam. Investigating an appropriate antifoam to minimize foam formation on the wastewater surface is recommended for future research [69]. The adhesion of clots on the electrodes' surface prevents coagulation ions' production. Therefore, it is recommended to immerse the electrodes in acid after each process and clean them completely for the next process [70]. The EC process generates less sludge but it may contain various toxic components, such as organic and inorganic substances, viruses, grease, and nutrients, which require proper control [71]. Additionally, It has been proved that high concentrations of chloride ions in landfill leachate can lead to chlorinated byproduct formation during EC. The use of boron-doped diamond electrodes can address this challenge [72]. Moreover, to optimize the EC process, it is necessary to manage the process parameters, such as pH, current density, electrolysis time, and inter-electrode distance [73].

## 4 Conclusion

This study evaluated the efficacy of the EC process in reducing leachate pollution potential concerning the variables of pH, current density, inter-electrode distance, and electrolysis time. RSM, CCD was used to design the experiments and optimize the treatment process. The main effect of the process factors and their interaction among each other was studied with the help of a statistical tool. Under optimal conditions of pH 8.0, 1.0 A/m^2^ current density, 0.5 cm inter-electrode distance, and 48 min electrolysis time, the EC method significantly decreased the pollution potential of leachate. The study showed a removal efficiency of 97.48 % for COD, 91.42 % for BOD_5_, 98.52 % for N-NH_3_, and 91.6 % for TDS. Moreover, the LPI index lowered from 38.06 to 7.22. The analysis of the kinetics of COD removal, an important factor in the LPI index, was also calculated. It was observed that COD removal follows the first-order model under optimum conditions. As a result, the EC process demonstrated significant potential for treating leachate and accomplishing discharge requirements.

## CRediT authorship contribution statement

**Fateme Ameli:** Formal analysis, Data curation. **Hassan Hashemi:** Writing – review & editing, Validation, Project administration, Data curation. **Mohammad Reza Samaei:** Writing – review & editing, Validation. **Esrafil Asgari:** Writing – review & editing, Project administration, Conceptualization. **Mehran Mohammadian Fazli:** Writing – original draft, Validation, Project administration, Methodology, Formal analysis, Data curation, Conceptualization.

## Declaration of competing interest

The authors declare that they have no known competing financial interests or personal relationships that could have appeared to influence the work reported in this paper.

## Data Availability

Data will be made available on request.
